# Injury of the Brain

**Published:** 1835

**Authors:** Philip J. Buckner

**Affiliations:** Georgetown, Ohio


### Art. V.—Injury of the Brain.

*Case of Fracture of the Cranium, with depressed bone, in which there was considerable loss of Brain, successfully treated.* By Dr. Philip J. Buckner.

On the 15th of January, 1833, I was called on to visit the son of Capt. Daniel Holden, of this county, a lad of about fifteen years of age. Eighteen hours previous he was thrown from a sleigh, the horse in full speed, and was dashed with his head against a tree. I found him in a perfectly comatose state—stertorous breathing—paralysis of the left side—cold extremities, with slow and feeble pulse—and dilated pupils. Had vomited several times during the night. I shaved the head, and found a large portion of depressed bone, covered by an extensive ecchymosis. I sent to Augusta, Kentucky, the nearest village, for Dr. Mackey, an aged and respectable physician, to assist me in the operation of trephining. Owing to unavoidable engagements he could not attend. I then, with the assistance of two of my pupils, Messrs. Rees and Woods, commenced an examination of the wound,—divided the integuments, and dissected up, so much as was necessary to ascertain the extent of the injury, and afford room for the application of the trephine, on firm bone.

The fracture was found to commence in the right orbit, at the inner canthus,—ran directly up the os frontis, to the coronal suture, following then the sagittal suture about an inch,—thence obliquely across the anterior third of the right parietal bone, crossing the squamous suture near the middle of the temporal bone, inclining forward through that bone, it terminated just behind the external canthus. The deepest depression was at the junction of the sagittal with the coronal suture. A portion of bone was removed with the trephine just below and in front of this point. It was now ascertained that the serrated edge of the depressed bone, had lascerated the dura and pia mater, and had separated a considerable portion of the cerebral substance. The coagulated blood, and detached brain, of the latter a table spoonful in quantity were removed. The depressed bone elevated,—the wound cleansed,—the parts approximated,—adhesive plaster and lint, with a roller, completed the dressing.

From the fracture's passing through the frontal sinus,—the great depression of bone,—and the fact, that the eyeball was thrown entirely from the socket, I am induced to believe the fracture extended some distance into the orbit. The patient having been placed in bed, I ordered warm frictions to the extremities,—a solution of sulphate of magnesia and tartarized antimony, in small and frequently repeated portions, together with stimulating enemas; with the view of establishing reaction, and counteracting the inflammation consequent upon so extensive an injury. I now left him, with directions that he should be bled, so soon as reaction should take place, provided the pulse became full and hard. The day after the operation I was taken ill, and was unable to visit him for four or five days, during which time he was visited once by a medical friend, (Dr. Taliaferro, of Washington, Kentucky,) who during my attack had visited me. He was also attended by Messrs. Rees and Woods, to whom, for their kindness and attention, both the patient and myself are much indebted.

On the 20th, I was able to visit my patient again,—found the wound beginning to suppurate,—tongue foul,—considerable delirium, and incoherent talking. Ordered 40 grains of calomel, followed by sulphate of magnesia, which brought off copious evacuations of dark bilious matter, with manifest relief. A considerable portion of the wound healed by the first intention, and its general appearance was favorable, until the eighth day after the operation, when an illconditioned abscess was discovered, just above the inner canthus of the eye, the consequence of a collection of matter in the frontal sinus, which, from the posture in which the patient lay upon his back, had been confined within the sinus, and became the source of much irritation.

About this time the febrile symptoms increased, he became restless, and complained much of his head and diseased eye, which was still greatly swollen and prominent, not having entirely receded within its orbit. Vision in this eye, so far as we could judge, was very indistinct, if not entirely lost, at this period. The pupil seemed permanently dilated, the surrounding integuments very livid, and the vessels of the ball and tunics were in a highly congested state. For the removal of the pus in the frontal sinus, I made a puncture through the integuments, covering the inner angle of the eye and supercilliary ridge, so as to penetrate the fracture, and pass into the frontal sinus, at its most dependent point. The matter thus dislodged, by the aid of cathartics and diaphoretics, the febrile symptoms subsided. The serrated edges of the fractured bone, immediately over the sinus, became *carious*, and were carried off by absorption, which continued along the course of the fracture, as high up on the os frontis, as the edge of the hair, and terminated in the opening made by the trephine. After this there was a general but gradual amendment. Nothing worthy of observation supervened, until the closing of the external wound. The absorption of bone, and that removed by the trephine, presents the singular appearance of a deep figure, running in a perpendicular line upon the forehead, of two and a half inches in length, and half an inch in width, through the whole length of which every pulsation of the brain may be distinctly seen and felt.

The condition of the eye continued to improve, during the healing of the external wound. It was about twenty days from the time he received the injury, before objects could be distinguished with the injured eye. Every thing for several days appeared inverted—as vision improved, objects appeared more distinct, but double, and at a greater distance than was real. Whether these phenomena were the result of inflammation, and a congested state of the blood vessels of the eye, or from an elongation, or shortening of the axis of vision, consequent upon the protrusion of the eyeball from its orbit; from the dilated state of the pupil, or from the too great or diminished sensibility of the optic nerve and retina, or whatever else, I leave as subjects of speculation, to the ingenious anatomist and speculative physiologist.

A question of some practical importance, however, might here be asked, Whether the fortuitous occurrence of the abscess in the frontal sinus, and consequent long continued drain of pus, so contiguous to the injured eye, was not a very great means of finally restoring the eye, and establishing perfect vision? The time, from the injury until the entire wound healed, was about thirty-five days; the part over the sinus being the last point closed. Sight, however, was not perfect until near a month after the wound healed. All that was done for the patient after the abscess ceased to discharge pus, was to give him for a short time tonics, and keep a pledget of lint, wet with a solution of muriat. ammo. to his eyebrow and forehead. He now enjoys perfect sight, and use of his intellectual faculties. It is near three years since the operation; the boy has had good health up to the present time, with the exception of two attacks, which it may not be improper to mention in connection with the foregoing history of the case.

The circumstances connected with each attack I think will not be uninteresting to my readers, as they appear intimately connected with, if not consequent upon, the previous injury of the head. During the month of August, about seven months after the operation, having worked very hard for several weeks, at harvesting, and hauling stone for the foundation of a mill, he was attacked with severe pain of the head, with chills and fever. He was supposed by his father, a very intelligent old gentleman, to have an attack of bilious fever, for which he gave him a portion of calomel, and followed it with a dose of ol. ricini, about which time he had a convulsion fit, and continued to have strong convulsions every twenty or thirty minutes, for several hours. I was immediately sent for. On my arrival he had had sixteen fits—was in a lethargic state—cold extremities—pallid countenance, and feeble pulse. I applied warm frictions, sinapisms, etc. to his extremities, and believing, from what the family told me, that the cause of his attack was from some undigested substance in his stomach, which, from irritation, had affected the head sympathetically, I gave him a gentle emetic, which operated three or four times, and brought off considerable food in an undigested state, and very sour. This was followed by a stimulating enema; after the operation of which the convulsions ceased, followed by intense arterial action. I now bled him freely, applied cold wet cloths to the head, kept up free catharsis by means of calomel and James' powder, followed by neutral salts. I left him much better; but in a few days, I was again sent for in haste, was informed by the messenger, "that the boy had been delirious all night, and talking incoherently, complained much of his head, that since daylight he had slept sound and could not be roused, that the family had discovered a large tumor or swelling on the forehead, over the open space occasioned by the loss of bone, and that the tumor could be seen to beat strongly." On my arrival I found the description given by the messenger, had not been exaggerated, but fell far short of the reality. He was catamose,—pupils dilated,—a tumor of the size of an egg, covering the artificial opening in the os frontis, and pulsating as strong and regular as the carotids themselves. A question would very naturally arise in the mind of the practitioner, Is it the cerebral substance protruding, or is it the collection of a fluid, and if a fluid, is it pus, blood, or serum? These are considerations which would produce some embarrassment in forming the diagnosis, as well as the proper course of treatment. From the symptoms, it was evident there was compression of the brain, from some cause. The tumor was firm, and regularly elastic. When firm pressure was made on it, it would recede within the cranium, but reappear immediately again, upon the pressure being taken off; and appeared to be attended with pain and restlessness. Upon a minute examination, I found the base of the tumor occupied a greater surface than the opening of the os frontis; the elasticity of its base being perceptible for some distance from the margin of the bone, surrounding the opening in the cranium. From these facts I was induced to believe that the tumor was produced by a fluid, which, insinuating itself between the integuments and bone, caused the fluctuation, which was perceptible at the base of the tumor, and larger in its circumference than the opening in the os frontis. Had it been produced by a protrusion of a portion of the cerebral substance, its base would have been circumscribed by the margin of bone through which it passed, and would have been rather less than the opening in the bone.

Feeling confident the young man must die, unless the pressure on the brain could be taken off, I determined on making an opening into the tumor, by a puncture with the lancet, when, to the gratification of all present, it was followed by a free discharge of perfectly transparent serum. It ran in a full stream until we caught about six ounces, after which the water continued to ooze from the orifice for about thirty-six hours, when it closed. In a few hours after this evacuation of water, the patient was restored to his senses, and convalesced rapidly. From the number of cloths wet by the fluid which oozed from the wound, it was believed by the family that the entire quantity discharged was not less than sixteen or eighteen ounces. The patient soon recovered, and continued to enjoy good health for eighteen months, when he was again seized with convulsions, accompanied with high febrile excitement, which had followed severe exercise. By prompt v. s., free catharsis, antimonials, and digitalis, the febrile symptoms were arrested, the convulsions subsided, and in a short time he was again restored to health.

In this last attack there was no tumor formed, except that during a convulsion there was a considerable prominence or convexity of the integuments covering the opening in the os frontis: affording conclusive evidence that, in convulsions, there is not only evident congestion of the brain, but actual enlargement of its volume, producing great irritability of the nervous system, with symptoms of compression of the brain; similar, though in a less degree, to those produced from a simple depression of bone; with this characteristic difference, that compression from depressed bone produces uniform and constant pressure, from the circumference to the centre; whereas compression from congestion of the vessels of the brain is from the centre to the circumference, and irregular in its application, from the convulsive systole and diastole of the heart and arteries; thus accounting, I think rationally, for the effusion, in the first attack. The exertion in the hot sun, of lifting and hauling heavy stone, produced a determination of blood to the head, and consequent congestion of the vessels of the brain, followed by convulsions and vomiting. The soft parts thereby being frequently thrust backward and forward through the opening in the os frontis, became irritated and inflamed by the rough edges of the cranium, and the inflammation terminated in effusion; which termination was prevented in the second attack by early and decisive anti-phlogistic treatment then employed. Since his recovery from this last attack he has had no return of disease. His mental faculties appear as good as they ever were, and so far as we can judge, he suffers no other inconvenience from the accident, than that occasioned from the permanent loss of bone from the forehead. PHILIP J. BUCKNER.

*Georgetown, Ohio, October*, 1835.

